# 

**Published:** 1900-01-13

**Authors:** 


**Notice to Advertisers and Subscribers.**

All Advertisements, Orders for Copies of The Hospital, and Business Communications should be addressed to **THE MANAGER (Not to the Editor)**, The Hospital, The Hospital Building, 28 & 29, Southampton Street, Strand, London, W.C.

The Cost of Subscription, post free, is as follows:—Per week, 2½d.; per quarter, 2s. 8d.; per half-year, 5s. 3d.; per annum, 10s. 6d. Foreign:—Per annum, 15s. 2d., payable, in all cases, in advance.

**NOTICE.—Vol. XXVI. of "The Hospital" (April, 1899, to September, 1899), in handsome cloth binding, is now on sale at the Office of this Journal, price 7s. 6d. Cases for binding the Numbers contained in this Volume 1s. 6d. Index to Vol. XXVI., 2½d., post free.**

**The Monthly Part for January is now ready. Price 6d., by post 8d.**

### BOOKS RECEIVED.

J. and A. Churchill.

"Effects of Borax and Boracic Acid on the Human System." By Dr. Oscar Liebreich.

Cornish Bros.

"The Two Disciplines in Education." An address by Sir William T. Gairdner, M.D., K.C.B., F.R.S.

William R. Jenkins, New York.

"Chemistry, its Evolution and Achievements." By Ferdinand G. Wiechmann, Ph.D.

C. Arthur Pearson.

"Home Nursing." By Sister Grace.

Chatto and Windus.

"Herbert Fry's Royal Guide to the London Charities." Edited by John Lane.

Baillière, Tindall, and Cox.

"Heart Disease: With Special Reference to Prognosis and Treatment." By Sir William H. Broadbent, Bart., M.D., F.R.S., F.R.C.P., and John F. H. Broadbent, M.A., M.D., M.R.C.P.

### The Student of Medicine.

### APPOINTMENTS.

W. ALLPORT, M.B., B.S.Lond., M.R.C.S.Eng., L.R.C.P.Lond., appointed Senior House Surgeon to the Birmingham and Midland Eye Hospital. A. Eddowes, M.D., M.R.C.P., appointed Honorary Physician to St. John's Hospital for Diseases of the Skin, Leicester Square. A. E. Giles, M.D., B.Sc.Lond., M.B., B.Ch.Vict., F.R.C.S.Edin., M.R.C.P.Lond., appointed Gynæcologist to the Tottenham Hospital. J. G. B. Gowlland, L.R.C.P.-Edin., L.R.C.S.Edin., L.F.P. & S.Glasg., appointed Resident Surgeon to the Clorcurry District Hospital, N. Queensland, Australia. E. Harries-Jones, M.B., C.M.Edin., appointed Resident Surgical Officer to the Birmingham and Midland Eye Hospital. G. P. Johnson, M.D.Glasg., D.P.H.Eng., appointed Medical Officer of Health to the Stoke-on-Trent Town Council. P. C. Maitland, M.R.C.S.Eng., L.R.C.P.Lond., &c., appointed Medical Officer to the Brompton and Knightsbridge Provident Dispensary. F. H. Pearce, M.R.C.S., L.R.C.P.Lond., appointed Senior Assistant Medical Officer to the Bradford Union Workhouse. S. Prior, M.B., C.M.Glasg., appointed Public Vaccinator of the Kirkheaton District of the Huddersfield Union. T. F. Roche, L.R.C.P., L.R.C.S.Edin., appointed Medical Officer for the Penarth District of the Cardiff Union. W. H. Rowlands, M.R.C.S., L.R.C.P.Lond., appointed Medical Officer of the Bromsgrove Union Workhouse. T. Savill, M.D., M.R.C.P., appointed Honorary Physician to St. John's Hospital for Diseases of the Skin, Leicester Square. A. F. Seacombe, L.R.C.P., L.R.C.S.Edin., appointed Resident Medical Officer to the Toxteth Workhouse. E. A. Worley, M.R.C.S., L.R.C.P.Lond., appointed Second Assistant Medical Officer to the St. Marylebone Infirmary.

Royal 8vo., illustrated by a series of Original Photo-micrographs and many Illustrations in the Text, cloth gilt, **7s. 6d.** net,

### THE MENOPAUSE AND ITS DISORDERS.

### With Chapters on Menstruation.

By A. D. LEITH NAPIER, M.D., M.R.C.P., late Editor of the *British Gynæcological Journal.*

"Of great practical use to the general practitioner as well as to those more specially engaged in gynæcological work."—*The Hospital.*

LONDON: THE SCIENTIFIC PRESS, LTD., 28 & 29, SOUTHAMPTON STREET, STRAND, W.C.

Crown 8vo., cloth gilt, 2s.

### DISTRICT NURSING ON A PROVIDENT BASIS.

By JAMIESON B. HURRY, M.D.

"A great deal of useful information will be found in this little book."—*Guardian.*

London: The Scientific Press, Ltd., 28 & 29, Southampton Street, Strand, W.C.

Accouchement Requisites.

Ambulance Cases.

### NOW READY,

A REPRINT OF

### A NURSING HANDBOOK

TOGETHER WITH S. MAW, SON, AND THOMPSON'S

### COMPLETE CATALOGUE OF NURSING REQUISITES.

The above work is now republished at a cost of 1s. per copy. Messrs. S. M., S., and T. have been compelled to make this charge on account of the extreme popularity of the work and the great expense incurred in its production.

***Post Free on receipt of 1s.***

### S. MAW, SON, and THOMPSON,

7 to 12, ALDERSGATE STREET, LONDON, E.C.

